# Cooperative Object Transportation Using Curriculum-Based Deep Reinforcement Learning

**DOI:** 10.3390/s21144780

**Published:** 2021-07-13

**Authors:** Gyuho Eoh, Tae-Hyoung Park

**Affiliations:** Industrial AI Research Center, Chungbuk National University, Cheongju 28116, Korea; gyuho.eoh@cbnu.ac.kr

**Keywords:** cooperative object transportation, curriculum, deep reinforcement learning, region-growing, policy-reuse

## Abstract

This paper presents a cooperative object transportation technique using deep reinforcement learning (DRL) based on curricula. Previous studies on object transportation highly depended on complex and intractable controls, such as grasping, pushing, and caging. Recently, DRL-based object transportation techniques have been proposed, which showed improved performance without precise controller design. However, DRL-based techniques not only take a long time to learn their policies but also sometimes fail to learn. It is difficult to learn the policy of DRL by random actions only. Therefore, we propose two curricula for the efficient learning of object transportation: *region-growing* and *single- to multi-robot*. During the learning process, the region-growing curriculum gradually extended to a region in which an object was initialized. This step-by-step learning raised the success probability of object transportation by restricting the working area. Multiple robots could easily learn a new policy by exploiting the pre-trained policy of a single robot. This single- to multi-robot curriculum can help robots to learn a transporting method with trial and error. Simulation results are presented to verify the proposed techniques.

## 1. Introduction

An object transportation technique using robots has been widely applied to diverse fields, such as logistics [1], exploration [2], a retrieval task [3], and service robotics [4]. Cooperative object transportation has been inspired by the collective behaviors of animals (e.g., ants) [5]. For example, ants can push or pull a large object that is much bigger than their bodies. They know by instinct that working together is better than alone. Inspired by this animal’s cooperative behaviors, many researchers have studied cooperative transportation techniques by imitating their actions. Some researchers presented a grasping method in which robots grasp an object using their manipulators and transport it to a goal [6]. Other researchers suggested a pushing method where robots push an object using their bodies [7]. A caging method is an extended pushing method by enclosing an object using multiple robots [8]. Although they have some advantages, there were many issues, such as requirements for the gripper, precise pushing control, and real-time acquisition of the object shape.

Recently, deep reinforcement learning (DRL)-based navigation techniques have made rapid progress. The DRL has been proven to be applied to various mobile robotics fields, such as collision avoidance, object transportation, multi-robot navigation, and social navigation [9,10,11]. Among them, DRL-based object transportation techniques have attracted attention from many researchers because DRL can solve tricky issues of conventional methods [12,13,14]. Using the DRL algorithm, robots can learn how to transport an object to a goal without preliminary knowledge. They do not have to consider complex interactive behaviors between robots and the object; they only need plenty of training data. Complicated or precise controls for object transportation are no longer necessary. However, it takes a long time to learn a transportation technique due to the necessity of sequential transportation procedures. For example, the typical process of object transportation is as follows [15]. First, multiple robots approach an object. Second, the robots prepare a proper formation for object transportation; they have to be heading toward a goal together. Finally, robots push the object to the goal after the previous processes are completed. These procedures should be executed in order, which are difficult to learn or require a long training time by random actions only.

Therefore, we propose a new cooperative object transportation technique using curriculum-based DRL. Multiple robots can learn an object transportation method by gradual learning from easy to difficult tasks; learned knowledge from easy tasks facilitates the learning of difficult tasks. We present two curricula based on this principle; *region-growing* and a *single- to multi-robot* curriculum. During the learning process, the pose initialization region is gradually extended according to the region-growing curriculum. If robots are proficient at transporting an object in a small region, the robots can easily learn a transportation method in a large region. The robots also exploit the previous knowledge that a single robot has already learned. Using this single- to multi-robot curriculum, robots can utilize the policy of a single robot to learn their new policies.

This paper is organized as follows. Section 2 presents related works with cooperative object transportation and DRL-based transportation. The object transportation problem is defined in Section 3. The preliminary knowledge for DRL-based object transportation is presented Section 4, and Section 5 presents the proposed DRL framework. Two curricula for object transportation are suggested in Section 6. Simulation results are presented in Section 7, and we discuss the importance of this paper in Section 8. Finally, our conclusions are given in Section 9.

## 2. Related Work

### 2.1. Cooperative Object Transportation

Cooperative object transportation methods are divided into three categories: grasping, pushing, and caging [16]. First, multiple robots can transport an object with a manipulator through grasping action [6,17]. The grasping method enables robots to manipulate an object precisely and robustly in a real environment. The movement of an object is restrained by robots with manipulators, which means that the object is under control by robots. However, the grasping method is not only intractable but also requires preliminary activity, such as object gripping. Additionally, the control complexity drastically increases as more robots are used because multiple robots should be controlled synchronously for object transportation. Second, multiple robots can push an object to a goal using their bodies [7,18]. In contrast to the grasping method, the pushing method does not require an equipped manipulator or proactive actions. Robots can change their poses freely because they are not tied to an object. However, the information of the surrounding environment, such as the static friction between an object and ground, object shape, or geometrical structures, should be known in advances. Finally, the caging method combines the advantages of robust manipulation in the grasping method and flexible object transportation in the pushing method [8,19,20]. Multiple robots approach an object and enclose it to prevent escape from robot formation. Then, robots can transport the object by maintaining the enclosing formation; robots do not have to consider the object’s movement during the transportation process. However, multiple robots should be precisely controlled to maintain the enclosing formation. In addition, an excessive number of robots is required for wrapping up a target object.

### 2.2. Deep Reinforcement Learning-Based Object Transportation

The main problem of conventional transportation methods is that predicting the movements of robots and an object is difficult. There are many unpredictable sensing and control errors between robots and an object in a real environment that can deteriorate the transportation capacity. Many researchers, therefore, have focused on learning-based object transportation methods to solve the problem. Wang and De Silva [21] presented a reinforcement learning-based cooperative box-pushing method. They showed that the performance of single-agent Q-learning was better than that of team Q-learning due to the lack of sufficient random actions. Rahimi et al. [13] compared single and multi-robot cases with RL-based approaches. They showed that the performance of cooperative box-pushing could be improved by frequent *Q-table* updates. The above-mentioned RL-based box-pushing methods are effective in simple and small environments; however, they cannot be applied in large environments, as high-dimensional spaces of state and action are necessary for describing large environments.

To overcome this problem, deep reinforcement learning (DRL) has received much attention in the machine learning field as an alternative to conventional RLs [22,23,24]. The most popular DRL application is Atari 2600 games with Google DeepMind [22]; the action-value function (Q-function) is approximated by a deep convolutional neural network called the deep Q-network (DQN). The DQN agent was able to surpass the performance of humans. Following that, various improved DRL methods have been presented, such as double Q-learning (DDQN) [25], deep recurrent Q-learning (DRQN) [26], and proximal policy optimization (PPO) [27]. With the aid of recent scholarly exploration of these DRL methods, many researchers attempted to apply DRL to the cooperative object transportation field. End-to-end reinforcement learning-based methods were presented for manipulating a large-sized object [12,28]. A decentralized DRL control scheme was presented for cooperative transport behavior [29], and a decentralized/centralized Q-net separation method for action selection was proposed [30]. In the field of animation, agent-based cooperative methods for pushing, pulling, and moving objects were studied [14].

The above methods, however, were operational only under specific conditions. For example, an object and robots should be connected with a rod in advance [29], transportation is only working in a grid environment [30]. In this paper, we focus on cooperative box-pushing with free-motion based on DQN, which enables this method to be applied to real environments without restrictions.

## 3. Problem Formulation

**Figure 1:**
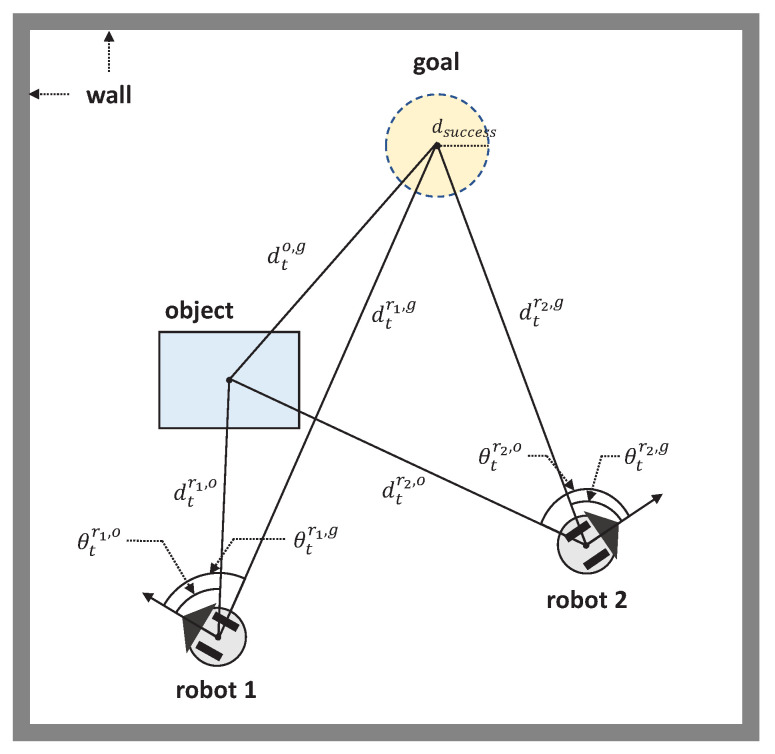
Object transportation problem description. Two robots transports an object to a goal. If the object is located at the goal within $d_{success}$, the object transportation is completed. The distance $d_{t}$ and angle $\theta_{t}$ between a robot and a target (an object or a goal) is represented with respect to the local coordinate of each robot.

The problem to be addressed in this paper is how to transport an object to the desired goal within a minimum time. Figure 1 shows the object transportation problem. Two robots are used to transport an object to a goal. An object cannot be transported by a single robot alone due to its heavy weight; however, two robots are able to transport the object by pushing together. If the object arrives at the goal within $d_{success}$, an object transportation is considered as *success*. On the contrary, object transportation fails if the object does not arrive at the goal during the maximum time steps.

We have four assumptions for the detailed problem formulation as follows. First, two-wheeled nonholonomic mobile robots are used for transportation on the Euclidean plane, and robot movements are partially restricted by kinematics and dynamics. Second, all robots have homogeneous characteristics. Third, we assume that all robots can identify the positions of an object and a goal with respect to each robot. This is a reasonable assumption because a robot can detect other objects using its own sensors, such as visual or LiDAR sensors. Finally, we assume that there are no obstacles and that each robot has the ability to recognize a target object. In reality, there are static and dynamic obstacles, and thus the ability to distinguish obstacles or objects is an important skill. However, we will concentrate on transportation methods in this paper; the detection method of static and dynamic obstacle is out of scope.

Therefore, the problem formulation can be described as follows:

(1) $$\begin{array}{cc} {\arg\min}_{\pi_{\theta }} & E[T|\pi_{\theta },\;s_{t}^{1},s_{t}^{2}],\\ \mathrm{subject}\mathrm{to} & \;\;\;d_{t}^{o,g}<d_{success} \end{array}$$

where *T* is the transportation time and $\pi_{\theta }$ is a policy that generates action *a* at time *t* given states $s_{t}^{1}$ and $s_{t}^{2}$: $a_{t}∽\pi_{\theta }\left(a_{t}|s_{t}^{1},s_{t}^{2}\right)$. The relative distance between an object and a goal is described as $d_{t}^{o,g}$. The object transportation succeeds when the distance between an object and the goal is less than $d_{success}$.

## 4. Preliminaries

### 4.1. Reinforcement Learning

Reinforcement learning (RL) is a kind of machine learning algorithm for how agents take actions to maximize accumulated returns in an environment [31]. The environment of RL can be described as a Markov decision process (MDP) which consists of the 4-tuples: (S,A,R,P). At each timestamp *t*, an agent observes a state $s_{t}\in S$ and selects an action $a_{t}\in A$ according to policy π, which is a mapping function from S to A. Then, a reward $r_{t}∼R\left(s_{t},a_{t}\right)$ is received, and the state $s_{t}$ is changed to $s_{t+1}∼P\left(s_{t},a_{t}\right)$. The agent executes this process iteratively until the terminal state is reached.

### 4.2. Deep Q-Learning

Q-learning is a model-free and off-policy reinforcement learning algorithm for predicting the long-term expected return [32]. This return is represented as a state-action value function $Q^{\pi }\left(s,a\right)$, which is an expected value by performing the action with the policy π given the state *s*. Choosing the action *a* to obtain a high Q-function Q(s,a) leads to better results than does a lower Q-function. The Q-function is iteratively updated by minimizing the loss between the current and expected Q-function as follows:

(2) $$Q\left(s_{t},a_{t}\right)\leftarrow Q\left(s_{t},a_{t}\right)+\alpha \left[r_{t}+\gamma \max_{a_{t+1}}Q\left(s_{t+1},a_{t+1}\right)-Q\left(s_{t},a_{t}\right)\right],$$

where α∈[0,1) is the learning rate, $r_{t}$ is the immediate reward, γ∈[0,1] is the discount factor, and $r_{t}+\gamma \max_{a_{t+1}}Q\left(s_{t+1},a_{t+1}\right)$ is the expected Q-function.

Initially, the Q-function was represented by a tabular method called a *Q-table*. The *Q-table* is sufficient to represent state and action space in a simple environment, such as a 5×5, 7×7, or 10×10 grid world. However, the real world should be represented by large-sized state and action space due to its complex and unstructured characteristics; the *Q-table* cannot only fully describe the real environment but also requires an extremely large memory.

For solving these problems, Mnih et al. [22] exploited a deep Q-network (DQN) instead of the *Q-table* for describing the Q-function. Thus, Equation (2) is rewritten with network parameters θ as follows:

(3) $$Q\left(s_{t},a_{t};\theta \right)\leftarrow Q\left(s_{t},a_{t};\theta \right)+\alpha \left[r_{t}+\gamma \max_{a_{t+1}}Q\left(s_{t+1},a_{t+1};\theta^{-}\right)-Q\left(s_{t},a_{t};\theta \right)\right],$$

where the $\theta^{-}$ are the parameters of target network, which is copied from θ. The Q-network is trained by minimizing the sum of differential loss functions $L_{i}\left(\theta \right)$ as follows:

(4) $$L_{t}\left(\theta \right)={\left[r_{t}+\gamma \max_{a_{t+1}}Q\left(s_{t+1},a_{t+1};\theta^{-}\right)-Q\left(s_{t},a_{t};\theta \right)\right]}^{2}.$$

In standard DQN, the training process is sometimes unstable, e.g., the Q-function can be drastically oscillated or varied during training. The core cause of unstable Q-function is time-correlation, which means that continuous state-action sequences have a negative effect on network optimization. Many researchers have attempted to solve this problem using the following methods. First, they extracted random sample batches from a data storage called *replay memory* [33] with a ϵ-greedy algorithm [34]; the time correlation between sample batches disappears using this random extraction method. Second, the parameters of the target Q-network, $\theta^{-}$, are periodically copied from θ, not all the time [22]; the frequent update of parameters makes the network unstable.

### 4.3. Curriculum Learning

Curriculum learning is a step-wise training strategy for efficient learning [35,36,37]. For example, when a teacher attempts to teach a new theory to students, the teacher helps the students to understand a basic concept first. If the students fully understand the basic concepts, then the teacher makes progress towards more difficult ones. Similarly, the students attempt to learn more difficult concepts if they are familiar with the previous difficult ones, and this is called curriculum learning.

Generally, an object transportation has a *sparse reward* problem; there is no reward until an object finally reaches a goal. For solving the sparse reward problem, we introduce two different curriculum approaches. The first is a randomized pose initialization in an extended region based on region-growing concept [38]. The second is a single- to multi-robot curriculum based on policy reuse [39]. Detailed explanations will be described in Section 6.

## 5. Reinforcement Learning Framework for Object Transportation

As already mentioned in Section 4.1, the MDP of an object transportation problem can be formulated as follows. First, the state of robot *i* at timestamp *t* consists of relative spatial information among a robot, an object, and a goal as follows:

(5) $$\begin{array}{c} s_{t}^{i}=\left[d_{t}^{r_{i},\;o},\;\cos\theta_{t}^{r_{i},o},\;\sin\theta_{t}^{r_{i},o},\;d_{t}^{r_{i},\;g},\;\cos\theta_{t}^{r_{i},g},\;\sin\theta_{t}^{r_{i},g}\right],\;\forall i\in \left[1,2\right], \end{array}$$

where $d_{t}^{r_{i},\;o}$ is the distance between a robot $r_{i}$ and an object, and $d_{t}^{r_{i},\;g}$ is the distance between a robot $r_{i}$ and a goal. The values $\theta_{t}^{r_{i},o}$ and $\theta_{t}^{r_{i},g}$ represent the angle differences between the robot heading $r_{i}$ and an object, and between the robot heading $r_{i}$ and a goal, respectively. The examples of states are shown in Figure 1. When two robots are used, a composite state $S_{t}$ is represented as $concatenate(s_{t}^{1},\;s_{t}^{2})$.

Second, a robot takes a position near an object and push it. Thus, the robot chooses an action $a_{t}^{i}$ for each timestamp *t*. For free robot motion, the action space consists of 6 actions as follows:

(6) $$a_{t}^{i}\in \left\{forward,\;backward,\;forward\;left,\;forward\;right,\;backward\;left,\;backward\;right\right\}.$$

Stop action is not defined because robots should move continuously until an object arrives at a goal; there is no need to use stop action for object transportation. The translational and rotational velocities of the actions in Equation (6) are presented in Table 1. In addition, a composite action space $A_{t}$ is introduced when multiple robots are used: $A_{t}=a_{t}^{1}\times a_{t}^{2}$.

Finally, a reward function should be designed for obtaining not only a final reward by reaching a goal but also consecutive rewards during the transport process. In typical object transportation methods, a final reward is provided only when an object reaches a goal. A robot rarely learns the transportation method if a reward is given once; which is known as a sparse reward problem. Therefore, we define a reward function for obtaining consecutive returns during the entire transportation process as follows:

(7) $$\begin{array}{c} r_{t}\left(s_{t},a_{t}\right)=\left\{\begin{array}{cc} 1 & \mathrm{if}\mathrm{an}\mathrm{object}\mathrm{reaches}a\mathrm{goal}\\ -0.01 & \mathrm{if}\mathrm{an}\mathrm{object}\mathrm{hits}\mathrm{the}\mathrm{wall}\\ -0.005 & \mathrm{if}a\mathrm{robot}\mathrm{collides}\mathrm{with}\mathrm{the}\mathrm{wall}\\ 0.1\times \Delta d_{t}^{r_{i},\;o}+0.5\times \Delta d_{t}^{o,\;g} & \mathrm{otherwise} \end{array}\right. \end{array}$$

where $\Delta d_{t}^{r_{i},\;o}=d_{t-1}^{r_{i},\;o}-d_{t}^{r_{i},\;o}$ and $\Delta d_{t}^{o,\;g}=d_{t-1}^{o,\;g}-d_{t}^{o,\;g}$. If a robot gradually approaches an object, the $\Delta d_{t}^{r_{i},\;o}$ has a positive value; it strengthens approaching action. On the contrary, if a robot moves further away from the object, the $\Delta d_{t}^{r_{i},\;o}$ has a negative value; it penalizes the leaving action. Similarly, the distance difference between an object and a goal, $\Delta d_{t}^{o,\;g}$, affects the selection of transport actions according to their spatial distance. We assigned a five-times larger weight to the distance difference between an object and a goal than that between a robot and an object. This is because the object transporting behavior to a goal is more important than the approaching behavior to an object. We defined the negative reward values according to the collision with a wall. The 100-times (object collision) and 200-times (robot collision) reward differences compared with the reward of reaching a goal were appropriate experimentally, as described in the second and third terms of Equation (7), respectively. More concretely, the recovery from an object stuck was more difficult than that of a robot stuck, and thus we gave double weight to the object hitting case.

## 6. Curricula for Efficient Learning

Learning for object transportation takes a long time to or sometimes fails to learn due to the sparse reward problem; a situation in which an object reaches the goal rarely occurs if guidance is not provided. To solve this problem, we introduced two curriculum-based learning methods: region-growing and single- to multi-robot curricula. Using the region-growing curriculum, robots can gradually learn their actions by extending the pose initialization region from a small to large region. In addition, multiple robots can learn a new complex task from a simpler task that a single robot has already learned, which is the single- to multi-robot curriculum. Detailed explanations will be addressed in the next sections.

### 6.1. Region-Growing Curriculum

In object transportation using RL, the success of learning highly depends on the positions where an object is initialized for each episode. For example, robots can learn how to transport an object if the object is initialized around a goal. This is because the traveled distance from an object to a goal is short, which enables robots to experience arriving a goal frequently. In the point of robots, object transportation is an easy task when an object is initialized near a goal. On the other hand, robots have difficulty transporting an object if the object is generated far away from a goal, which is a difficult task. Long traveling distances cause robots to fail transportation. However, if robots are fully experienced with easy tasks in advance, robots can succeed in object transportation even when an object is initialized far away from a goal. This is a core principle of the region-growing curriculum.

**Figure 2:**
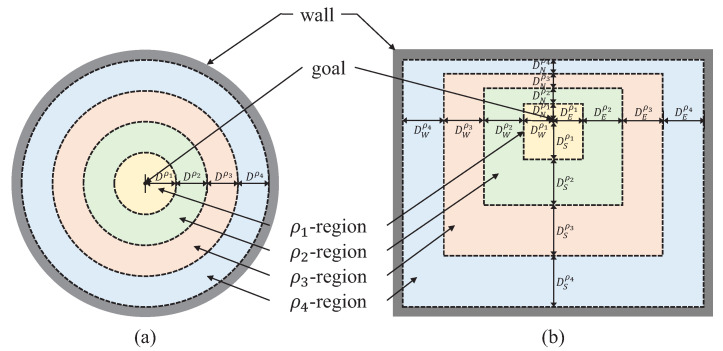
The region partitioning examples of region-growing curriculum. An environment is uniformly partitioned into $\rho_{j}$-region (j=1,2,⋯,H) from a goal; *H* is 4 in this figure. (**a**) A circular environment can be divided using a circle with an identical interval: $D^{\rho_{1}}=D^{\rho_{2}}=D^{\rho_{3}}=D^{\rho_{4}}$. (**b**) A rectangular environment with asymmetrical shapes can be partitioned into difference intervals $D_{k}^{\rho_{i}}$ (k∈{E,S,W,N}).

Based on this principle, we adopted the region-growing concept for setting the stage for learning. Region-growing is a kind of image segmentation method [38] that partitions an image by extending a region from a seed point. We adjusted the degree of learning difficulty using the region-growing technique. For example, we allowed an object to be initialized in the $\rho_{1}$-region only, as illustrated in Figure 2. In this case, robots can learn how to transport the object, which was initialized in the $\rho_{1}$-region only. If robots are good at object transportation in the $\rho_{1}$-region, then an allowable region for pose initialization is extended from $\rho_{1}$ to $\rho_{2}$. Similarly, robots can learn how to transport an object in the $\rho_{2}$-region. This process continues until the whole region is covered: $\rho_{1}\to \rho_{2}\to \rho_{3}\to \rho_{4}$. Robots can gradually learn how to transport an object by extending regions in which the object is initialized. The region shape can be determined differently according to an environment: a symmetrical and circular partitioning (Figure 2a) and an asymmetrical and rectangular partitioning (Figure 2b).

In this section, we consider a single robot only for concentrating on the effect of region-growing curriculum learning. The region-growing curriculum is also useful when multiple robots are applied. We will address a multi-robot region-growing case including the single- to multi-robot curriculum in the next section. Algorithm 1 presents the process of region-growing curriculum for single-robot DQN. First, we initialize the replay memory D and Q-networks for active and target (lines 1–3). Second, we set an initialized region according to the number of episodes using the region-growing algorithm (lines 5–7); the more the episodes increase, the more the initialized region extends. The rest of the algorithm is similar to a typical DQN [22]; select and execute action $a_{t}$ with ϵ-greedy algorithm, and then observe $s_{t+1}$ and store multiple transitions $\left(s_{t},a_{t},s_{t+1},r_{t+1}\right)$ into D for whole steps (lines 8–14). Third, train an active Q-network with random mini-batches using a gradient descent method (lines 15–17). Finally, we update the target Q-network with the active Q-network for *K* episodes (line 18).
**Algorithm 1:** Region-growing curriculum-based single-robot DQN.
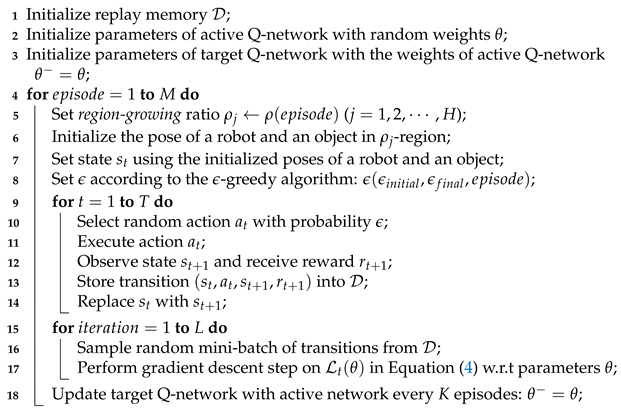


### 6.2. Single- to Multi-Robot Curriculum

Multi-robot object transportation is a more complex task than a single-robot case because robots should manipulate an object by cooperative behavior; robots should not only perform their own transport actions but also consider the actions of other robots. In addition, they should simultaneously push the object in the same direction to a goal. Taking such actions by the random exploration of multiple robots is almost impossible.

Robots, however, can learn a cooperative transport technique if they reuse the policy already learned by a single robot; which is called policy-reuse [39]. The single- to multi-robot curriculum is based on the policy-reuse method, and the core idea of this curriculum is that a complex problem (i.e., multi-robot object transportation) can be solved using prior knowledge (i.e., a transporting method by a single robot). Multiple robots can concentrate on cooperative behaviors because basic transporting actions have already been learned by a single robot.

**Figure 3:**
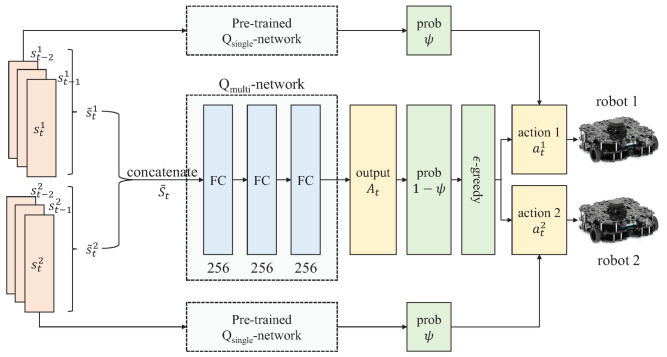
$Q_{multi}$-network training procedure. The states of the two robots (${\tilde{s}}_{t}^{1}$ and ${\tilde{s}}_{t}^{2}$) are concatenated with one state (${\tilde{S}}_{t}$) and are used as an input for a $Q_{multi}$-network. Three consecutive states of each robot are considered as one state: ${\tilde{s}}_{t}^{i}=\left\{s_{t-2}^{i},s_{t-1}^{i},s_{t}^{i}\right\}\left(t\geq 2\right)$ for i∈{1,2}. The $Q_{multi}$-network consists of three fully-connected (FC) layers. In the beginning of training, each robot selects their actions from the pre-trained $Q_{single}$-network according to with a probability of ψ. The probability ψ gradually decreases from 1.0 to 0.0, and thus, an output action set $A_{t}$ from ${arg max}_{A_{t}}Q_{multi}\left({\tilde{S}}_{t},A_{t}\right)$ is used for selecting actions with a probability of 1−ψ. The action set $A_{t}$ is divided into two actions (i.e., $a_{t}^{1}$ and $a_{t}^{2}$) for each robot.

Figure 3 shows how to train a multi-robot $Q_{multi}$-network using the pre-trained single-robot $Q_{single}$-network. Each robot takes an action using the pre-trained $Q_{single}$-network with a probability of ψ and $Q_{multi}$-network with a probability of 1−ψ; robots reuse the pre-trained $Q_{single}$-network for selecting their actions and train a $Q_{multi}$-network using multiple transitions generated by the $Q_{single}$-network. The probability ψ gradually decreases from 1.0 to 0.0, which means that they gradually use their $Q_{multi}$-network to take their actions as episodes proceed. Actions from the pre-trained $Q_{single}$-network help robots to induce transport behaviors. They succeed in transporting an object from the beginning, even if there is a non-stationary environment. The input of the $Q_{multi}$-network is a concatenated state ${\tilde{S}}_{t}$ with three consecutive states of each robot. Output action $A_{t}$ is partitioned into two actions ($a_{t}^{1}$ and $a_{t}^{2}$) for giving an order to each robot.

Algorithm 2 presents the single- to multi-robot curriculum-based DQN. First, we initialize a replay memory and parameters of $Q_{multi}$-network, and load the pre-trained $Q_{single}$-network in advance (lines 1–4). The region-growing curriculum is also applied for multi-robot object transportation (lines 6–7). Using the $Q_{single}$-network with a probability of ψ, each robot can take approaching and transport actions from the beginning (lines 12–15). With the help of the actions by $Q_{single}$-network, multiple robots can train their network $Q_{multi}$ (lines 16–20). The rest of the algorithm is similar to that of the region-growing curriculum (Section 6.1) except the concatenation of states.
**Algorithm 2:** Single- to Multi-Robot Curriculum-Based DQN.
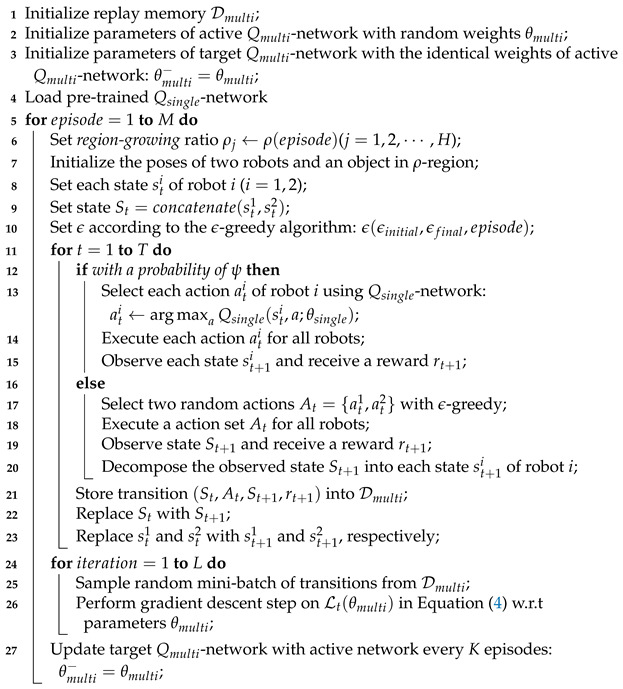


## 7. Results

We conducted simulations to verify our proposed algorithms. First, we will describe a simulation environment and hyper-parameters in Section 7.1. Second, region-growing curriculum-based transportation results using a single robot will be presented in Section 7.2. Third, a single- to multi-robot curriculum-based transportation results based on policy-reuse will be described in Section 7.3. Finally, we tested the proposed method in a environment with action noise to verify robustness in Section 7.4.

### 7.1. Simulation Environment

**Figure 4:**
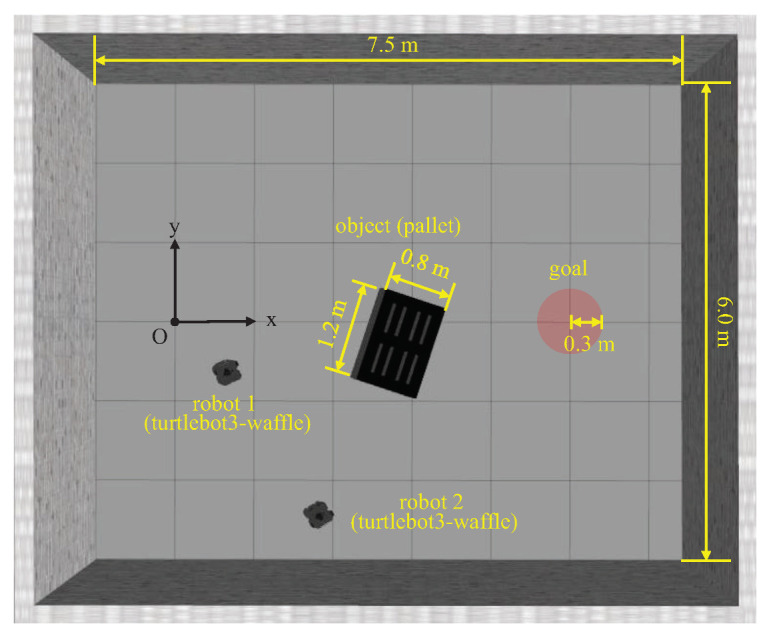
Simulation environment constitution using Robot Operating System (ROS) gazebo. We assumed that two robots transport a pallet to a goal in a warehouse. We set the mass of a pallet as 0.1 and 0.3 kg for the single-robot and multi-robot experiments, respectively. A single robot can push a lightweight pallet where the mass is 0.1 kg but cannot push a heavyweight pallet where the mass is 0.3 kg. Object transportation is considered as success when a pallet is located within the boundary of the goal by 0.3 m.

We constituted a simulation environment, as shown in Figure 4. We assumed a warehouse scenario in which one or two robots transported a pallet (i.e., object) to a desired point (i.e., goal). The total size of the workspace was 7.5 m (W) × 6.0 m (H) and that of the pallet was 1.2 m (W) × 0.8 m (H). The radius of the goal is 0.3 m, and each grid in the figure represents 1 m. The position of the goal was fixed as (5.0, 0.0) m, but that of the pallet and robots were randomly initialized for each episode.

**Figure 5:**
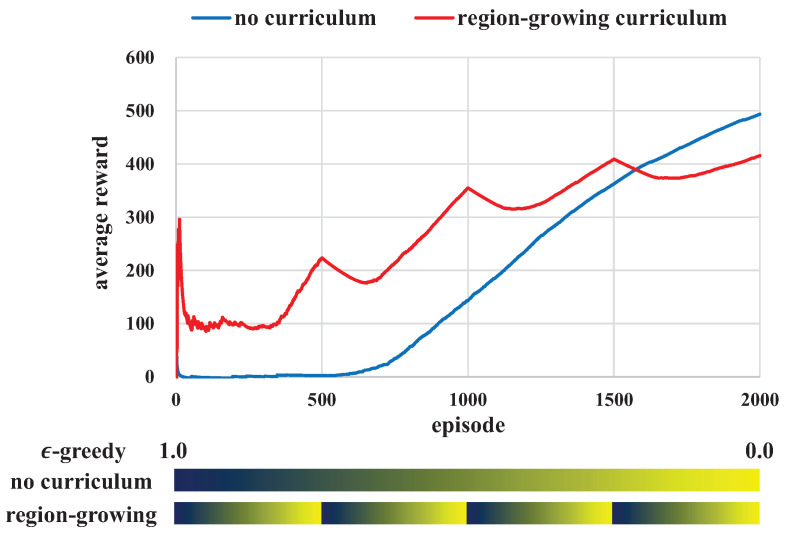
Average reward graphs of the region-growing curriculum and no-curriculum. The average rewards of the region-growing curriculum show increase and decrease curves at regular intervals due to the iterative changes of ϵ. In the region-growing curriculum, an ϵ-greedy algorithm should be applied at regular intervals because a robot should first explore at a new region. In the bar graph, the blue and yellow colors represent ϵ is 1.0 and 0.0, respectively.

Turtlebot3-waffle [40] was used as a transporter robot, and the diameter of the robot was about 0.3 m. A gazebo with ROS noetic was used to constitute the simulation, which makes a physics model based on an open dynamics engine (ODE). We played Gazebo simulations about 70 times fast, and it took about 10 and 20 h to train 2000 (single robot) and 4000 (multi-robot) episodes, respectively; these total training numbers of episodes were experimentally determined because the average reward curve converged from those of episodes, as will be described in Figure 5. The training was conducted on Geforce GTX-1650 and Intel i7-9700 3.00 Ghz. The hyper parameters of training are described in Table 2.

### 7.2. The Results of Region-Growing Curriculum

We conducted simulations to verify the performance of the region-growing curriculum. For concentrating on the effect of the region-growing curriculum, we only consider a single robot in this section; the case of multiple robots will be addressed in the next section. We set the mass of the pallet as 0.1 kg, which is light enough to be manipulated by a single robot. We determined that the whole region is uniformly partitioned into four divisions based on a goal: *H* equals 4 in Algorithm 1, and distance ratios among $\rho_{j}$-regions are identical, as shown in Figure 2b.

Figure 5 shows the average rewards for the no-curriculum and region-growing curriculum. Initially, the average rewards showed little improvement due to the exploration duration regardless of curriculum; the value of ϵ given by the ϵ-greedy algorithm was high in the initial period, which was 1.0. The average reward of the no-curriculum increased continuously as episodes proceeded.

However, the average reward of the region-growing curriculum showed increases and decreases at regular intervals because ϵ was changed periodically in the region-growing curriculum. For example, ϵ was 1.0 at episode 0 and ϵ decreased continuously until the number of episodes equaled 500. Then, the value of ϵ was initialized to 1.0 again when the episodes were 500. This process was executed iteratively until total episodes ended. Although there was a small difference, the region-growing curriculum-based learning showed larger average rewards than the no-curriculum learning until 1600 episodes. This means that the curriculum-based RL can be readily trained from the beginning; the pose initialization in a restricted small region raises the probability of transport completion. The average reward of the no-curriculum overtook the region-growing curriculum after about 1600 episodes. However, this result did not affect the success rate as shown in Table 3; the region-growing curriculum helped a robot to learn a transportation method for the whole region, not for specific regions.

The success rate of the region-growing curriculum was 5% higher than no-curriculum as described in Table 3. In addition, the average traveling distance of the region-growing curriculum was shorter than that of the no-curriculum, and the average steps for transportation completion were also smaller than that of the no-curriculum. This means a robot can learn a transportation method with small energy consumption.

### 7.3. The Results of Single- to Multi-robot Curriculum

Unlike the previous section, we used two multiple robots and a heavyweight pallet for the simulation of the single- to multi-robot curriculum. We set the mass of a pallet as 0.3 kg, which is too heavy to be transported by a single robot alone, but two robots can push the pallet with cooperative behavior. The pre-trained network of a single robot, $Q_{single}$-network, was generated by the region-growing curriculum of a single robot in Section 7.2. In addition, we tested not only the single- to multi-robot but also the region-growing curriculum to verify the overall performance of curriculum DRL-based object transportation methods.

**Figure 6:**
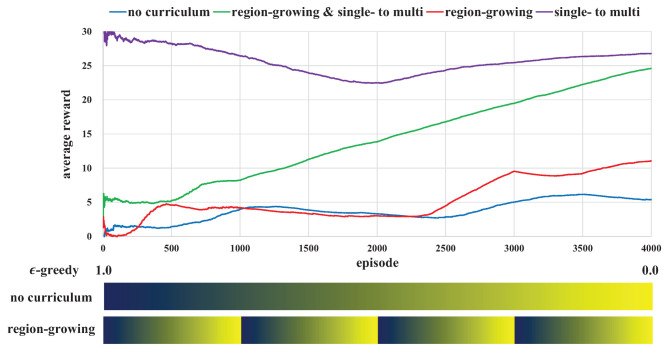
The average rewards according to the combinations of region-growing and single- to multi-robot curricula. The single- to multi-robot curriculum was highly affected by the performance of object transportation; robots could not obtain a large reward without the single- to multi-robot curriculum.

Figure 6 shows the average reward according to the combinations of curricula. The average reward of the single- to multi-robot curriculum was the largest but did not always increase during episodes. On the other hand, the average reward continuously increased when both single- to multi-robot and region-growing curricula were applied. The sustained growth of the average reward indicates that the robots kept learning the object transportation method. Although the average reward of the two curricula was lower than that of the sole single- to multi-robot curriculum, the success rate of the two curricula was the highest as described in Table 4. The average reward rarely increased when neither the region-growing nor the single- to multi-robot curricula were applied; which indicates that the robots could not learn anything. Robots experienced rare success because they did not receive any guidance for object transportation learning, which dropped the possibility of obtaining a reward. The average reward of the single- to multi-robot curriculum was higher than that of the region-growing curriculum, which means that the application of the pre-trained model was more helpful than restricting the pose initialization area.

The success rate of the multi-robot object transportation methods are summarized in Table 4. We also compared the curriculum-based and the previous multi-robot Q-learning methods called *independent Q-learning* (IQL) [41]. For all cases, the success rates of the two robots case was lower than that of a single robot case due to the change of the problem situation (i.e., pallet mass: 0.1 kg → 0.3 kg at Section 7.3). The success rate was the highest when both the region-growing and single- to multi-robot curricula were used. On the other hand, the success rates were close to zero when the single- to multi-robot curriculum was not applied, which means that the pre-trained policy using a single robot provided crucial chances for the success of object transportation. The performance of IQL was also poor because the strategy of cooperative behavior could not be learned by the IQL.

**Figure 7:**
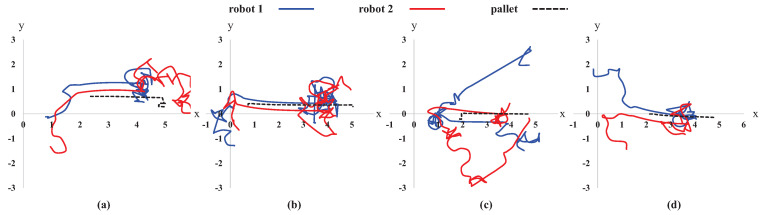
Sample paths of object transportation by two robots. The positions of the robots and a pallet were randomly initialized in each case; however, the goal pose was always (5.0, 0.0) m. (**a**) When the pallet was positioned around a goal, robots attempted to adjust the direction of the pallet by changing their positions. Robots 1 and 2 attempted to push the pallet from behind and front, respectively. (**b**) There was sometimes excessive unnecessary behaviors at the vicinity of a goal. (**c**,**d**) If two robots were initialized at a long distance, two robots attempted to approach a pallet first and then pushed it to a goal.

The sample paths of multi-robot transportation using the region-growing and single- to multi-robot curricula are shown in Figure 7. Two robots gathered around a pallet if the robots were initialized at different positions. Then, they pushed the pallet to a goal by maintaining a line formation. There were sometimes unnecessary behaviors while robots approached and transported the pallet; robots showed redundant actions, especially at the vicinity of the goal.

### 7.4. Results in a Environment with Action Noise

We also tested the proposed methods by adding noise to verify the effect of control errors. Gaussian noise with a zero-mean and increasing standard deviation σ was added to action values as follows:

(8) $$\begin{array}{cc} v_{noise}^{i} & =v^{i}+N\left(0,\sigma_{v}^{i}\right)\\ \omega_{noise}^{i} & =\omega^{i}+N\left(0,\sigma_{\omega }^{i}\right) \end{array}$$

where $v^{i}$ and $\omega^{i}$ are the translational and rotational velocities without the noise of robot *i*, respectively. Table 5 shows the success rate in a environment with action noise from N(0,1.0) to N(0,5.0). At first, the success rate slightly decreased until σ was 1.0. However, the success rate drastically decreased from σ=2.0, and it was closed to zero from σ=3.0. Although the proposed method was robust to small noise, it could not guarantee performance if the action noise exceeded a specific threshold.

## 8. Discussion

To solve the cooperative object transportation problem, two main issues should be addressed. The first is how to deal with sequential tasks, such as the approaching and transport phases; these phases should be executed in order. Second, multiple robots should be fully cooperative with synchronized motions, which means they should push an object together to manipulate an object. The DRL could be a solution to these issues because of its effective learning ability; however, it cannot be applied directly due to a sparse reward problem. Therefore, we presented region-growing and single- to multi-robot curricula in this paper.

First, the region-growing curriculum made the problem easier by restricting the initialized region of an object. The region-growing curriculum can be applicable to diverse environments regardless of environment size and obstacle distribution; if we design the region-growing steps appropriately, this curriculum could be exploited in various practical fields, such as warehouses, factories, and garbage collection centers. Second, the single- to multi-robot curriculum helped robots to work together based on the pre-trained policy of a single robot. The pre-trained policy provides a rough learning direction of multiple robots, which means that complex and cooperative behaviors of multiple robots can be learned from the simple behavior of a single robot. The two strategies have learning with steps in common; the object transportation method is gradually learned from easy to difficult tasks.

However, there are certain limitations to our work. First, the diverse shapes of objects were not validated. We only tested the proposed method using a rectangular object, i.e., a pallet, because we assumed a warehouse scenario. In reality, there are various shaped objects, and thus we should also consider these objects. We, therefore, will study various-shaped object transportation using DRL in future work. Second, the proposed methods are inappropriate to use more than two robots (i.e., N≥3), because the dimensions of the state and action spaces exponentially increase as more robots are used. For example, if we use six actions as the same as in this paper, the dimensions of the action space will be $6^{10}$ when 10 robots are used; this dimension is unlikely to be manageable using the proposed DRL framework. Meanwhile, there is another solution for using multiple robots; a distributed multi-robot team can be applied to object transportation. If we introduce the distributed system, each robot can decide actions based on its own sensing information. The state and action spaces are always identical regardless of the number of robots because a robot considers its own status. Even in this case, however, there is also a learning problem due to the characteristics of a non-stationary environment; the policies of other robots are continuously changing according to learning steps. In the future work, we will examine an extensible cooperative object transportation system by considering the non-stationary characteristics. Finally, practical experiments were not conducted. Although robots were tested in the simulation environments with action noise assuming real experiments, the simulation to real transfer performance (i.e., Sim-to-Real) should be verified. We left the Sim-to-Real verification as future work.

## 9. Conclusions

In this paper, we proposed two curriculum-based reinforcement learning methods for object transportation: the region-growing and single- to multi-robot. The region-growing curriculum extended the initialization region of an object, and the single- to multi-robot curriculum used the pre-trained policy of a single robot for cooperative object transportation. Both curricula had a common principle that complicated and difficult tasks could be learned from easy tasks. If robots are proficient in easy tasks, then the difficult tasks can be solved. Based on these curricula, robots can overcome the learning fail problem of deep reinforcement learning. We also tested the proposed methods in an environment with action noise and assuming a real environment. The proposed methods can be applied to various fields, such as foraging, logistics, and waste collection.

## Figures and Tables

**Table 1:** The translational and rotational velocities of actions in Equation (6).

Action	v (m/s)	ω (rad/s)
*forward*	1.0	0.0
*backward*	−1.0	0.0
*forward left*	1.0	0.3
*forward right*	1.0	−0.3
*backward left*	−1.0	0.3
*backward right*	−1.0	−0.3

**Table 2:** Hyper-parameters of reinforcement learning.

Parameters	Symbol in Algorithm 2	Value
discount factor	γ	0.999
learning rate	α	0.001
initial exploration prob	$\epsilon_{initial}$	1.0
final exploration prob	$\epsilon_{final}$	0.1
batch size	-	512
replay buffer size	# of $D_{multi}$	$1.0\times 10^{7}$
update period of $Q_{target}$	*K*	20 episodes
partitioned number of regions	*H*	4
total number of episodes	*M*	2000 (single), 4000 (multi)
maximum step size	*T*	1000
iteration number of training	*L*	100 (single), 200 (multi)

**Table 3:** The region-growing curriculum result of a single robot.

Performance Index		No Curriculum	Region-Growing Curriculum
success rate (success/total)		0.91 (181/200)	0.96 (192/200)
average travelled distance	pallet	92.89 m	71.72 m
	robot	162.99 m	139.04 m
average steps per episode		59.00	51.57

**Table 4:** The success rate of the multi-robot object transportation methods.

Method	Success Rate (Success/Total)
IQL [41]	0.01 (2/200)
DQN without curriculum	0.02 (4/200)
Region-growing curriculum	0.045 (9/200)
Single- to multi-robot curriculum	0.36 (72/200)
Region-growing and Single- to multi-robot curricula	0.77 (154/200)

**Table 5:** The success rate in a environment with action noise.

Noise	Success Rate (Success/Trial)
No noise	0.770 (154/200)
N(0,1.0)	0.620 (124/200)
N(0,2.0)	0.140 (28/200)
N(0,3.0)	0.045 (9/200)
N(0,4.0)	0.040 (8/200)
N(0,5.0)	0.010 (2/200)

## Data Availability

Not applicable.
